# Pattern-induced visual discomfort and its cumulative effects revealed by pupillary measures

**DOI:** 10.3389/fnhum.2025.1723675

**Published:** 2026-01-07

**Authors:** Ron Meidan, Yoram S. Bonneh

**Affiliations:** 1 School of Optometry and Vision Science, Faculty of Life Sciences, Bar-Ilan University, Ramat Gan, Israel; 2 Gonda Multidisciplinary Brain Research Center, Bar-Ilan University, Ramat Gan, Israel

**Keywords:** cumulative effects, pattern glare, pupillary response, spatial frequency, visual discomfort

## Abstract

**Introduction:**

Viewing repetitive striped patterns can induce pattern glare, experienced as visual discomfort (VD). While previous studies examined either pupillary responses or VD separately, few have investigated how they covary or evolve with repeated exposure. This study tested whether pupillary dynamics could serve as potential physiological indicator of individual visual sensitivity beyond subjective reports.

**Methods:**

Across four experiments (preliminary: *n* = 97; main: *n* = 70 for spatial frequency, *n* = 46 for central field size, *n* = 36 for central blank, with partial overlap), we manipulated spatial frequency, central field size, and surround field size of square-wave gratings (0.5–3 s) while measuring both discomfort and pupil size.

**Results:**

Higher spatial frequencies and larger pattern areas elicited stronger pupillary constriction and greater discomfort, whereas repeated exposures produced cumulative increases in discomfort and decreases in baseline pupil size, consistent with visual strain rather than adaptation. To assess the potential of pupillometry as an indicator of visual discomfort, we examined individual differences in the main spatial-frequency experiment (controlled viewing distance, *n* = 42). A paradoxical pattern emerged: within participants, stronger stimuli produced greater constriction, but individuals with higher overall discomfort showed weaker constriction and stronger late redilation. Similar dissociations between subjective sensitivity and pupillary responses have been noted in studies of light-induced discomfort, suggesting that related mechanisms may contribute, although their specific physiological basis remains unclear.

**Discussion:**

Overall, our findings clarify how pattern-induced discomfort evolves over time and across individuals and highlight pupillometry's potential as a sensitive, physiological tool for assessing visual sensitivity.

## Highlights

- Striped patterns systematically increased discomfort and pupillary constriction
- Repeated exposure led to progressive discomfort and baseline pupil size reduction
- Among high-sensitivity participants, weaker constriction and stronger redilation appeared
- The paradox may reflect interindividual autonomic differences under visual stress
- Pupillometry shows promise as a potential physiological indicator of visual sensitivity

## Introduction

Visual discomfort (VD) refers to an unpleasant sensory or perceptual experience triggered by specific visual inputs, including high-contrast patterns, flickering lights, and repetitive geometric structures. Such stimuli deviate from the statistical properties of natural scenes and can evoke symptoms such as eyestrain, headaches, nausea, or perceptual distortions.

Among the most widely studied triggers of VD are striped patterns, particularly black-and-white square-wave gratings. These patterns contain sharp luminance transitions and multiple spatial harmonics (Campbell and Robson, 1968), and they closely resemble real-world visual inputs such as lines of text, striped fabrics, and architectural elements (Wilkins et al., 2007). Early work by Wilkins et al. (1984) showed that such patterns reliably induce discomfort and perceptual distortions in susceptible individuals. O'Hare and Hibbard (2011) demonstrated that visual noise with a natural 1/f amplitude spectrum (typical of natural images) was the most comfortable, whereas any deviation from this natural spectrum by adding excess energy increased discomfort. These findings align with the design of the Pattern Glare Test (Evans and Stevenson, 2008), which assesses pattern-related visual stress using gratings at 0.5, 3, and 12 cpd.

In addition to evidence linking stripe patterns to reading-related visual discomfort (Wilkins and Nimmo-Smith, 1987; Renckens, 2024), heightened sensitivity to striped patterns has also been documented in several clinical populations, including migraine (Shepherd et al., 2013; Hine and White, 2021), autism spectrum conditions (Torrens et al., 2024), and chronic pain (Ten Brink and Bultitude, 2022). In these groups, assessments often rely on subjective reports, which may be challenging for children or individuals with communication limitations. This motivates the development of complementary objective measures.

Pupillometry offers one such measure. The pupil responds to luminance, and pupillary dynamics include melanopsin-mediated contributions (Lucas et al., 2003; Spitschan et al., 2017). However, the pupil also responds systematically to spatial structure (Barbur and Thomson, 1987), and discomfort-inducing visual inputs can evoke stronger pupil constriction (Lin et al., 2015; Ayzenberg et al., 2018). Patterned stimuli can elicit a spatially driven pupillary constriction, known as the Pupil Grating Response (PGR), in which the pupil constricts at stimulus onset even when mean luminance remains constant (Barbur and Thomson, 1987; Hu et al., 2019).

Despite extensive work on pattern-induced VD and separate research on pupillary responses, few studies have examined how specific stripe-pattern spatial attributes jointly influence subjective discomfort and pupil behavior. Across four experiments, we manipulated spatial frequency and pattern field size of square-wave gratings while measuring both VD and pupillary responses. Our first aim was to determine how these pattern attributes influence discomfort and pupil size and to characterize the relationship between the two. Our second aim was to examine how visual discomfort and pupil responses evolve across repeated exposures. Finally, we assessed whether pupillary responses could serve as an objective marker of visual sensitivity, potentially complementing subjective reports in populations with communication challenges, such as young children.

## Materials and methods

### Participants

In the preliminary experiment, which consisted of two separate parts (eye-tracking and subjective rating), 97 adults participated in total (89 in the eye-tracking part, of whom 12 also completed the subjective rating part, plus 8 participants only in the subjective part). In Experiment 1 (spatial frequency), 70 adults participated. In Experiment 2 (central field size), 46 adults participated. In Experiment 3 (central blank), 36 adults participated (noting that certain participants took part in more than one of these main experiments). The overall gender distribution was nearly balanced (106 males, 111 females). Participant age was restricted to 18–35 years to minimize age-related variability in pupil size. Sample sizes (*n* = 36–70) were determined pragmatically based on participant availability, yet yielded high statistical power given the large effect sizes observed (partial η^2^ > 0.14). All participants had normal or corrected-to-normal vision, were recruited following routine vision examinations, and were screened for any history of epilepsy or migraine. Experiments were conducted on different days without specific pre-experiment restrictions (e.g., no instructions regarding caffeine intake, screen exposure, or sleep patterns were imposed). Some participants completed more than one experiment though the majority participated in only one experiment. No familiarization session was provided beyond the first trial of each experimental block.

The experiments received approval from the Bar-Ilan University Institutional Review Board (IRB) Ethics Committee. Written informed consent was obtained from all participants, and all procedures adhered to IRB guidelines.

### Apparatus

Pupil size was recorded using a Tobii 4C eye tracker with a sampling rate of 90 Hz. The preliminary experiment utilized the tracker mounted on a 15.6-inch laptop monitor, while subsequent experiments employed a Yoga 7 14ITL5 laptop featuring a 14^′′^ FHD IPS display (1920 x 1080). To ensure consistent display conditions, screen luminosity was set to maximum values, and automatic luminosity adjustments were disabled across all experiments. We directly measured the luminance of the basic display elements using a Minolta LS-100 device. Measurements for full background field yielded: white = 274 cd/m^2^, brown (Experiment 1) = 21 cd/m^2^, and black = 0.08 cd/m^2^. These measurements were taken at the standard viewing distance of 75 cm.

Stimulus presentation and data collection were managed through PSY, an in-house developed platform for psychophysical and eye-tracking experiments previously used and validated in several of our studies (e.g., Bonneh et al., 2015). No chin rest was used, to simulate field-deployable conditions suitable for diverse populations including children, with viewing distance continuously measured by the eye tracker. Exploratory *post-hoc* analyses further examined subsets of participants based on their measured viewing distance, in order to assess whether accommodation or screen luminance at shorter distances might confound individual-level correlations. Although viewing distance was continuously tracked, it was only used in *post-hoc* analyses to identify individual differences (e.g., restricting participants to the 75–85 cm range) and was not enforced during data collection. The effective monitor visual angle was approximately 22.5° × 13.2° at the standard 75 cm viewing distance corresponding to the approximate average measured in the experiments. Eye tracking was conducted binocularly, with analyses focused on the left eye data. The visual discomfort (VD) rating scale ranged from 0 (no discomfort) to 5 (maximum discomfort), conceptually similar to established measures such as the Visual Discomfort Scale (Conlon and Hine, 2000) and the Leiden Visual Sensitivity Scale (Ten Brink et al., 2021). No explicit training was provided; instead, the first trial served as a familiarization trial for each participant.

### Stimuli and procedure

#### Design rationale

All patterned stimuli were high-contrast square-wave gratings (Michelson contrast ≈ 1) following the Pattern Glare Test (Wilkins et al., 1984). Such gratings reliably induce visual discomfort and a transient Pupil Grating Response (PGR) even at constant luminance, providing an established probe of spatial-pattern sensitivity in both typical and clinical populations. To dissociate spatial-structure effects from luminance-driven reflexes, the experiments systematically varied spatial frequency (Experiment 1) and pattern size (Experiments 2–3) while measuring mean screen luminance: (1) Experiment 1: varied spatial frequency at fixed mean luminance (10.5 cd/m^2^; brown background); (2) Experiment 2: increased patterned-field size on a white background, decreasing mean luminance (274 → 137 cd/m^2^); (3) Experiment 3: enlarged central white window within a patterned surround, increasing mean luminance (137 → 214 cd/m^2^). Thus, Experiment 2 and 3 provided complementary luminance manipulations: Experiment 2 decreased luminance by expanding the patterned area, while Experiment 3 increased it by enlarging the unpatterned region. Together, these manipulations helped ensure that any observed pupil and discomfort effects were not artifacts of illumination but reflected genuine sensitivity to spatial patterning. Presentation durations (500–3,000 ms) were optimized in pilot testing to elicit reliable pupil responses while minimizing fatigue.

#### Preliminary experiment

Black-and-white square-wave patterns were presented at spatial frequencies of 0.10, 0.33, 0.69, 1.64, and 7.20 cycles per degree. At the lowest frequency (0.10 cpd), the stimulus consisted of a single wide black bar occupying most of the central visual field, producing the lowest central luminance among all patterned conditions. At higher frequencies, the stimulus transitioned from a single bar to alternating black–white stripe pairs, increasing the amount of white visible within the stimulus itself, although all stimuli remained darker than the uniform white inter-stimulus screen.

The upper limit of 7.20 cpd was determined based on pilot testing and physiological considerations. Research indicates that pattern-induced visual discomfort typically peaks at mid-range spatial frequencies; specifically, individuals with high visual discomfort report peak symptoms around 4 cpd, while responses in less sensitive groups may extend to 8–12 cpd (Conlon et al., 2001; Evans and Stevenson, 2008). This subjective profile parallels the pupillary response, which demonstrates bandpass characteristics with maximal constriction occurring within the 2–8 cpd range (Barbur and Thomson, 1987; Hu et al., 2019).

Luminance values for the display elements were 0.08 cd/m^2^ for black and 274 cd/m^2^ for white. Each stimulus was displayed for 3,000 ms, followed by a 3,000 ms full-field white screen, with each condition repeated five times in random order. Visual discomfort (VD) ratings and pupil measurements were collected in two separate sessions: an automated eye-tracking session and a separate VD-rating session in which each new stimulus appeared only after the participant provided a response.

#### Experiment 1—spatial frequency

Stimuli consisted of full-screen square-wave gratings (21.10° × 12.70°) at spatial frequencies of 0.36, 0.47, 0.73, 1.72, and 4.97 cpd, comprising black stripes (0.08 cd/m^2^) on a brown background (normalized RGB 0.39, 0.25, 0.09; ≈ RGB 99, 64, 23; luminance 21 cd/m^2^). At all spatial frequencies, only stripe width varied while the 50:50 ratio of black to brown remained fixed, yielding constant mean screen luminance of 10.5 cd/m^2^.

The brown background was selected based on a pilot study comparing multiple background colors at fixed grating frequency. Brown produced the lowest visual discomfort and smallest pupil constriction, minimizing ceiling effects and improving sensitivity to spatial-frequency effects and individual-level correlations. White backgrounds were used in Experiments 2–3 to maximize luminance dynamic range as pattern size varied (see Design Rationale).

Each stimulus was shown for 2,500 ms, followed by a full-field brown inter-stimulus interval (21 cd/m^2^) lasting a minimum of 1,500 ms (extended until the participant provided their discomfort rating via button press), yielding an average inter-trial onset interval of approximately 5s. Each condition was repeated three times in random order, and pupil size was continuously tracked throughout the session. Viewing distance varied slightly around the instructed 75 cm, as estimated by the eye tracker.

#### Experiment 2—central field size

This experiment examined the effect of the central patterned area while keeping spatial frequency constant. Previous work by Gao et al. (2020) demonstrated that pupil constriction increases systematically with the size of equal-luminance gratings, even when overall luminance is held constant, indicating that pupil responses are influenced by spatial structure as well as brightness.

Here, black-and-white square-wave gratings (stripe width ≈ 0.15°, spacing ≈ 0.10°, spatial frequency ≈ 4 cpd) were presented on a white background (274 cd/m^2^). The visible pattern area varied from 0.08° × 0.08° (approximately 0.006 deg^2^, < 0.01% of screen) to 21.10° × 12.70° (approximately 268 deg^2^, effectively filling the entire 268 deg^2^ display). Within each patterned region, black and white pixels maintained a 50:50 ratio (mean pattern luminance ≈ 137 cd/m^2^), while unpatterned regions remained white (274 cd/m^2^). As pattern size increased, mean screen luminance decreased systematically. Stimuli were presented for 500 ms, followed by a minimum inter-stimulus interval of 1,500 ms, extended until participants provided their VD rating. Each condition was repeated three times in random order, with continuous pupil recording throughout.

#### Experiment 3—surround field size

Stimuli consisted of a full-field square-wave grating (spatial frequency ≈ 4 cpd) extending over the entire display (21.10° × 12.70° ≈ 268 deg^2^), with central blank windows revealing the white background underneath. Blank window sizes ranged from 3.10° × 3.10° (approximately 9.6 deg^2^, 3.6% of screen) to 12.10° × 12.40° (approximately 150 deg^2^, 56% of screen). A control condition displayed the complete pattern without a blank window. The patterned surround maintained a 50:50 black-white ratio (mean ≈ 137 cd/m^2^), while the blank window revealed only white (274 cd/m^2^). As the blank window enlarged, mean screen luminance increased systematically. Stimulus timing and rating procedures were identical to those in Experiment 2, and each condition was repeated three times in random order.

### Data preprocessing

Pupil size data was analyzed to remove blinks and artifacts, following our previous (Kadosh et al., 2024). Blinks were detected when pupil size dropped to zero, and their exact onset and offset were refined using vertical eye movements exceeding 4 SD from baseline (calculated from the first third of the analysis window). We examined periods spanning 100 ms before and 150 ms after each blink. Blink windows outside the 250–750 ms range were marked as missing data, often due to head movement. On average, approximately 25% of the data was missing due to blinks and tracking issues, which were partly due to free head and participant movement.

#### Epoch extraction and pupil size modulation

**Figure 1:**
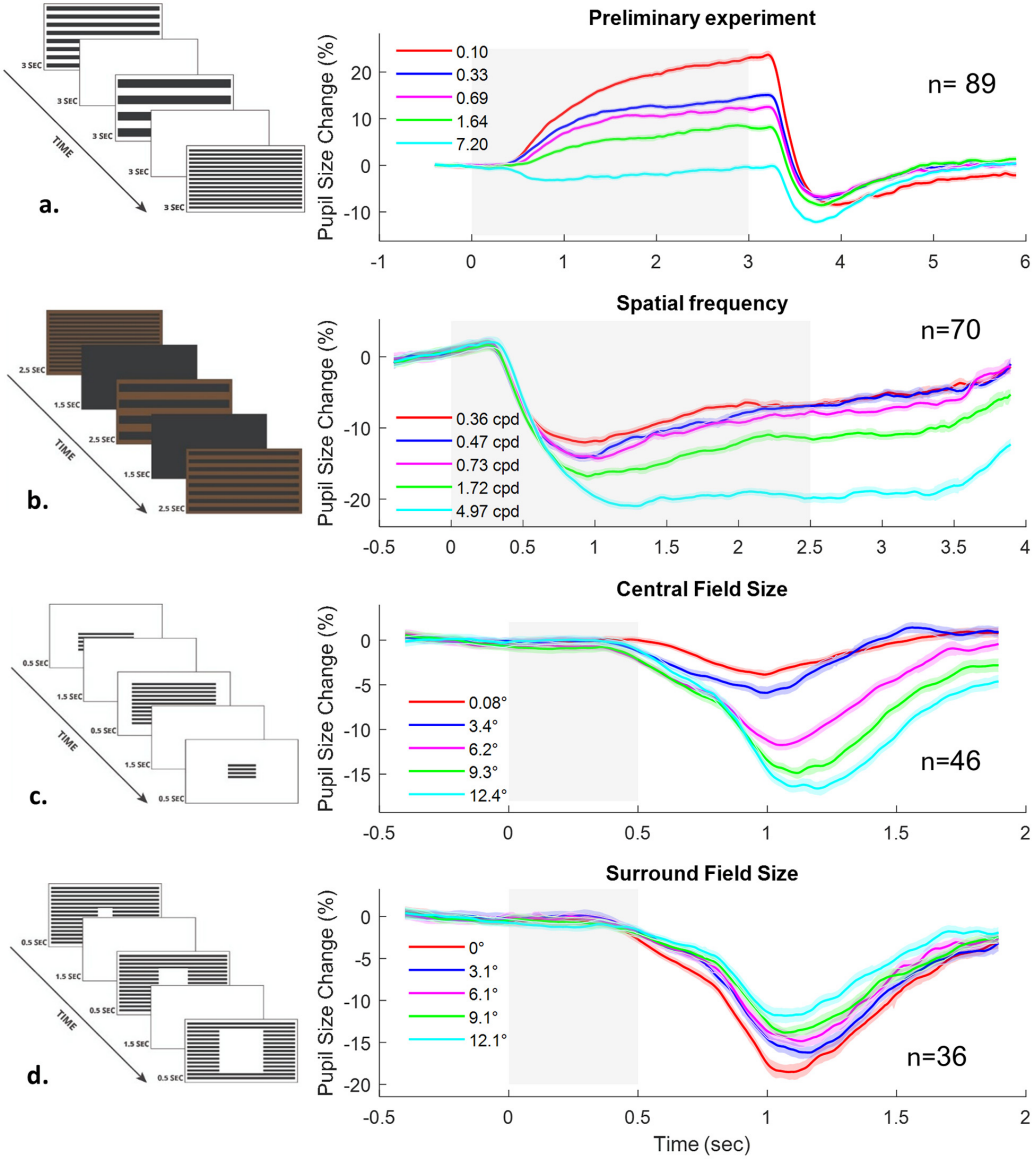
Pupillary responses to striped patterns across four experiments. Left: Square-wave gratings used in each experiment. Right: Corresponding pupillary time courses (gray shading indicates stimulus duration). **(a)** Preliminary experiment: Gratings with varying spatial frequencies (0.10–7.20 cpd) on a white background (3,000 ms on, 3,000 ms off), shown either automatically or with discomfort ratings. **(b)** Experiment 1: Similar gratings (0.36–4.97 cpd) on a brown-green background (2,500 ms on, 1,500 ms black screen) to reduce discomfort. **(c)** Experiment 2: Gratings with varying field sizes (1.0°-12.4°), shown for 500 ms, followed by a 1,500 ms white screen. **(d)** Experiment 3: Gratings with different central blank sizes (0.1°-9.1°), same timing as **(c)**. All experiments recorded pupil size and visual discomfort. Stripe luminance was constant (0.08 cd/m^2^); backgrounds were white (274.3 cd/m^2^) or brown (Exp. 1). The screen samples in a, b are similar to the actual stimulus with scaling, while the spatial-frequency shown in c, d was reduced to 30% for clarity.

We extracted stimulus-aligned epochs spanning 0.5 s before onset to 3 s after offset for pupil size and eye position. To compute pupil size modulation (Figure 1), traces were normalized to the pre-onset average. Outliers (>2 SD per time point) were excluded within participants, and the data were then averaged across participants. Error bands represent ±1 standard error.

#### Discrete measures extraction

For all experiments, we extracted baseline pupil size (pre-stimulus) and constriction responses. Baseline pupil size was defined as the average diameter during the 0.5 s preceding stimulus onset. Constriction was defined as the minimum pupil size (% change relative to baseline) within the 0.5–1.5 s window after stimulus onset, capturing the main constriction phase. In the preliminary experiment, constriction was instead measured in a 1–2.5 s window, to obtain initial estimates. In the main spatial-frequency experiment (Experiment 1), we additionally examined redilation, defined as the maximum pupil size (% change relative to baseline) in the 2–3 s post-stimulus recovery window.

### Statistical analysis

Statistical analyses were conducted using both Linear Mixed Models (LMM) and repeated-measures ANOVA to assess the effects of experimental manipulations on pupillary responses and visual discomfort ratings.

#### Linear mixed models

Linear trends were assessed using linear mixed-effects modeling implemented in MATLAB (Hohenstein et al., 2017). The LMM approach was chosen for its ability to handle repeated measures, missing data, and continuous predictors while accounting for both fixed and random effects. Separate LMMs were fitted for each experiment and dependent variable. Fixed effects included the experimental factors of interest, and random effects included participant (random intercept) to account for between-subject variability. Models were fitted using maximum-likelihood estimation. Typical model specifications were of the form:

*lme* = *fitlme(tbl, 'y* ~ *frequency* + *(1|participant)‘);* for experiments with a single continuous predictor, or *lme* = *fitlme(tbl, 'y* ~ *condition* ^*^ *frequency* + *(1|participant)');* when both categorical and continuous factors were included. The significance of each fixed effect, including continuous predictors, was evaluated using MATLAB's anova(lme) function, which provides an *F*-test for each term in the model. For each fixed effect, we report the regression coefficient (β), standard error (SE), *t*-statistic, degrees of freedom, and *p*-value.

#### Repeated-measures ANOVA

To complement the linear mixed-effects (LMM) analyses and to provide standardized effect-size estimates, repeated-measures ANOVAs were conducted for experiments involving categorical factors (e.g., stimulus condition). In contrast, frequency was modeled as a continuous predictor within the LMM framework, yielding a single slope parameter per experiment. For each ANOVA, we report the F statistic with degrees of freedom, *p*-value, and partial eta-squared (η^2^) as a measure of effect size.

## Results

### The effect of pattern glare on pupil size and visual discomfort ratings

In the preliminary experiment, participants viewed striped gratings at different spatial frequencies. The study included 89 participants in the eye-tracking session and 20 in the separate subjective rating session, with 12 completing both sessions (*n* = 97 unique participants). Pupil size was expressed as percentage change from baseline (Figure 1a). Because the background was white, the pupil dilated overall but also showed the Pupil Grating Response (PGR), a reflexive constriction linked to cortical processing of patterns that increased systematically with spatial frequency, consistent with previous reports (Barbur and Thomson, 1987; Hu et al., 2019). VD ratings, collected in a separate complementary session without eye tracking, also increased with spatial frequency, while showing the same trend as the pupillary responses even though they were measured in different sessions.

In the main experiments, pupil size and VD ratings were measured simultaneously. The experiments manipulated three stimulus attributes: spatial frequency (Experiment 1), central field size (Experiment 2), and surround field size (Experiment 3). As shown in Figure 1, pupil constriction varied with stimulus characteristics: greater constriction occurred with higher spatial frequency (Exp. 1, Figure 1b), with larger central field size (Exp. 2, Figure 1c), and with larger surround field size (Exp. 3, Figure 1d; note reversed colors). Average viewing distances were 75.7 cm (SD = 7.2), 72.3 cm (SD = 9.9), and 76.3 cm (SD = 12.3) for Experiments 1–3, respectively.

**Figure 2:**
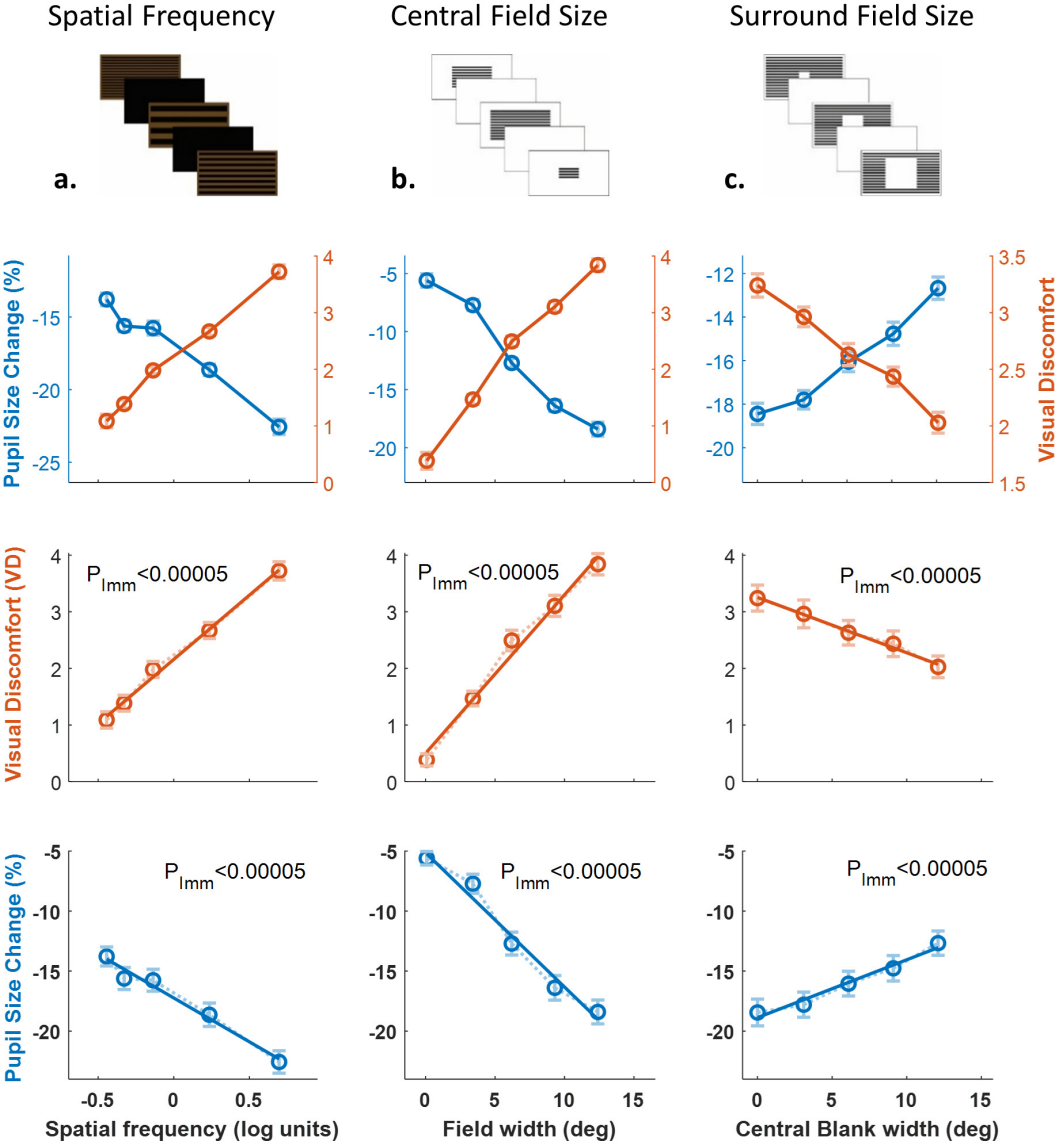
Systematic relationships between experimental variables and visual responses across three experimental paradigms. **(a)** Combined visualization of visual discomfort and pupillary responses. Visual discomfort (VD) ratings (orange circles, right y-axis) and pupillary constriction responses (blue circles, left y-axis) plotted against experimental variables. Left: Spatial frequency (Experiment 1, *n* = 70), Middle: Central field size (Experiment 2, *n* = 46), Right: Central blank size (Experiment 3, *n* = 36). Both measures demonstrate systematic and opposite trends: as visual discomfort increases, pupillary constriction becomes stronger (more negative values). **(b)** Visual discomfort ratings plotted separately against each experimental variable. All relationships were statistically significant (P_lmm_ < 0.00005), demonstrating systematic increases in subjective discomfort with increasing spatial frequency, field size, and decreasing central blank size. **(c)** Pupillary constriction responses plotted separately against each experimental variable. All relationships were statistically significant (P_lmm_ < 0.00005), showing systematic changes in objective pupillary responses that mirror the subjective discomfort patterns. Data show means ± 1 SE.

Figure 2 illustrates systematic relationships between stimulus variables and visual responses. In Figure 2a, pupillary constriction (blue, left axis) and VD ratings (orange, right axis) are plotted together across the three manipulations: spatial frequency (Exp. 1, *n* = 70), central field size (Exp. 2, *n* = 46), and central blank size (Exp. 3, *n* = 36). Data were averaged within participants (with outlier exclusion, see Methods), then across participants, with ±1 SE error bars computed on demeaned data (with subject means subtracted to remove between-subject variability and better reveal within-subject effects of experimental manipulations).

Both measures showed monotonic, approximately linear relationships: pupil constriction increased (more negative change), whereas VD ratings increased with stimulus intensity. Figures 2b, c shows each variable separately (VD and pupil constriction), using identical y-axis ranges for comparison across experiments. Significance was tested with linear mixed models (LMMs; see Methods). All six analyses (VD ratings and pupillary responses across Experiments 1–3) yielded *p* < 0.00005 in the overall F-tests from the LMMs (anova(lme)), confirming highly significant linear relationships. Detailed LMM statistics are provided in Table 1. To assess effect sizes, we also conducted repeated-measures ANOVAs for each experiment (Table 2).

**Table 1:** LMM statistical detail for Figure 2.

**Experiment**	**β VD**	**Pupil**	**SE VD**	**Pupil**	**t VD**	**Pupil**	**df VD**	**Pupil**	**p VD**	**Pupil**
Exp 1	67.79	−1.99	2.09	0.16	32.36	−12.78	1,063	975	1.8 × 10^−160^	1.1 × 10^−34^
Exp 2	85.39	−3.39	2.99	0.15	28.57	−22.67	703	670	9.9 × 10^−120^	7.2 × 10^−85^
Exp 3	−34.08	1.24	2.37	0.15	−14.37	8.24	896	687	2.8 × 10^−42^	8.6 × 10^−16^

**Table 2:** One-way ANOVA statistical detail for Figure 2.

**Experiment**	**Measure**	**F(df_1_, df_2_)**	***p***	**partial η^2^**
Exp 1	Pupil	F(4, 345) = 14.2	*p <* 9.8 × 10^−11^	0.14
Exp 1	VD	F(4, 345) = 56.5	*p <* 1 × 10^−36^	0.40
Exp 2	Pupil	F(4, 225) = 38.5	*p <* 1.5 × 10^−24^	0.41
Exp 2	VD	F(4, 225) = 70.5	*p <* 1.3 × 10^−38^	0.56
Exp 3	Pupil	F(4, 249) = 4.0	*p <* 3.7 × 10^−3^	0.06
Exp 3	VD	F(4, 285) = 9.0	*p <* 7.8 × 10^−7^	0.11

The lowest degrees of freedom for the pupil constriction compared to VD (F-test df = 249 compared to 285) was due to missing eye-tracking data in different conditions for 7 of the 58 participants. All effects for experiments 1–2 demonstrated large effect sizes (η^2^ > 0.14), while the effects of experiment 3 were “medium”.

### The relationship between visual discomfort and pupillary measures

**Figure 3:**
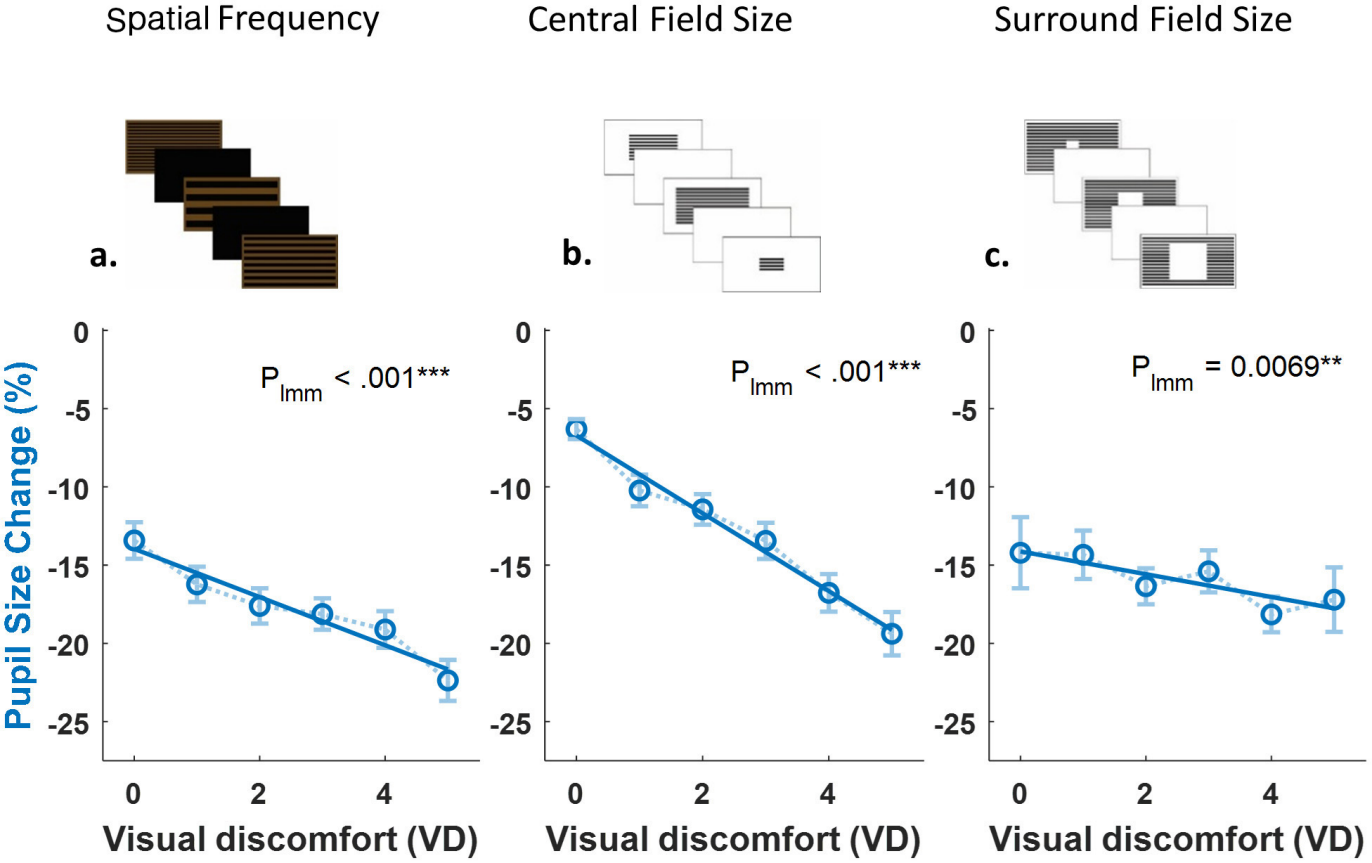
Relationships between visual discomfort ratings and pupillary measures across three experimental conditions. Graphs showing the relationship between visual discomfort (VD) ratings and pupil size change (%) across three experiments manipulating different stimulus properties: **(a)** Spatial frequency (70 participants); **(b)** Pattern field size (46 participants); **(c)** Central aperture size (36 participants). In all three conditions, increased visual discomfort was associated with greater pupil constriction (Spatial frequency: P_lmm_ < 0.001; Pattern field size: P_lmm_ < 0.001; Central aperture size: P_lmm_ = 0.0069). Each data point represents mean pupil response for participants who provided that specific VD rating level (0–5 scale). The number of participants contributing to each specific rating level varied (41–59 for spatial frequency, 29–43 for field size, 15–28 for aperture size) because participants provided repeated measures (rating multiple stimuli) and may not have used every possible rating level (0–5 scale). Linear mixed-effects models (LMM) accounted for repeated measures within participants.

Following the demonstration that both VD and pupil constriction systematically vary with stimulus characteristics (Figure 2), we examined their direct correlation independent of specific stimulus parameters (Figure 3). To this end, we combined all stimulus conditions and plotted average pupil change (percentage from baseline) for each VD rating (0–5). This analysis allowed us to test whether subjective discomfort and pupillary responses are linked across different manipulations.

The LMM analyses results are shown in Table 3.

**Table 3:** LMM statistical detail for Figure 3.

**Experiment**	**β**	**SE**	***t***	**df**	***p***
Exp 1	−2.02	≈ 0.16	−12.39	975	7.2 × 10^−33^
Exp 2	−2.48	≈ 0.15	−16.86	667	2.2 × 10^−53^
Exp 3	−0.59	≈ 0.2	−2.93	687	0.004

These findings demonstrate that subjective reports of visual discomfort are systematically associated with objective pupillary responses. Importantly, the correlations emerged when collapsing across conditions, showing that the link holds independently of specific stimulus attributes.

### Cumulative effects of visual discomfort and pupillary responses

**Figure 4:**
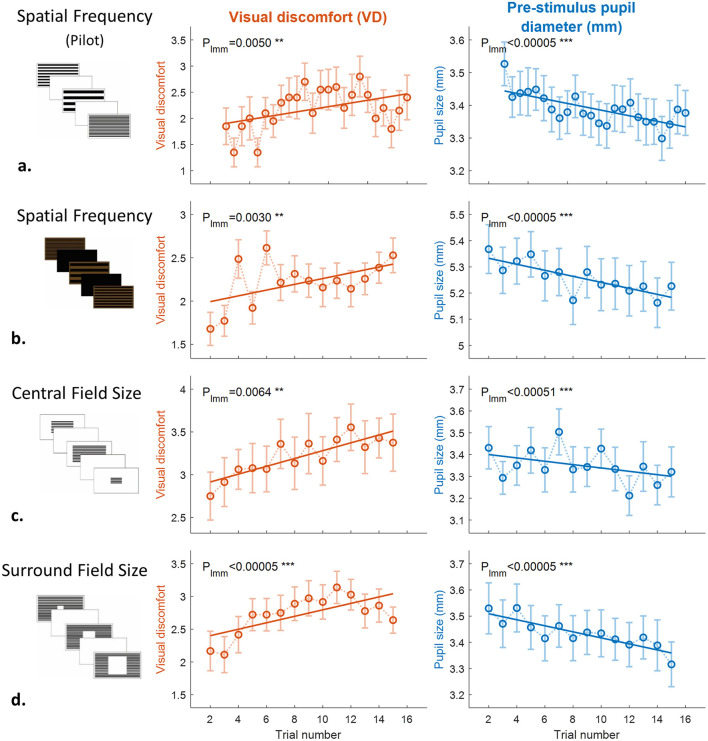
Cumulative effects of visual discomfort and pupil diameter across experiments. Visual discomfort (VD) ratings (left column, orange) and pre-stimulus pupil diameter in mm (right column, blue) as a function of trial number for: **(a)** preliminary spatial frequency with *n* = 20 for VD and n = 89 for pupil in separate sessions, **(b)** spatial frequency with *n* = 70, **(c)** central field size with *n* = 46, and **(d)** central aperture with *n* = 36. The first trial in each experiment was excluded from analysis as it served to familiarize participants with the procedure. In central field size, the two smallest field conditions were excluded from analysis due to near-floor VD ratings. For pupil data, analyzed sample size per trial varied due to quality filtering: 75–85 in preliminary; 54–66 in spatial frequency; 22–32 in central field; 30–36 in central aperture. VD ratings increased across trials in three experiments; central field showed significance at *p* = 0.0064. Pupil diameter decreased across trials in all experiments (*p* < 0.0005). Error bars: SEM.

Figure 4 shows how VD ratings (left column) and pre-stimulus pupil size in mm (right column) changed across repeated exposures. The first trial in each experiment served as familiarization and was excluded from analysis. Across subsequent trials, VD ratings progressively increased in three of four experiments, reflecting an accumulation of visual discomfort with repeated stimulation. In the central field size experiment, significance was obtained only after excluding the two least aversive conditions (very small central fields, e.g., 0.08°), which consistently produced near-floor VD ratings. Removing these conditions allowed the analysis to focus on stimuli capable of eliciting measurable discomfort and revealed a clear cumulative effect (*p* = 0.0064 vs. 0.55; Figure 4c). For the pupillary data, relative constriction (percentage change from baseline) did not show the expected accumulation across repeated exposures. This likely reflects the fact that baseline pupil size itself gradually shifted across trials, making relative measures less sensitive to cumulative changes. We therefore analyzed the pre-stimulus baseline pupil size in mm, which provided a clearer indicator of trial-dependent accumulation effects.

Significance was tested with linear mixed models (LMMs; see Methods). All eight analyses revealed significant trial-by-trial increase of VD ratings and decrease of pupil size across the pilot and Experiments 1–3, with *p* < 0.007 (5 of 8 with ^***^) in the overall F-tests from the LMMs confirming highly significant linear relationships. The details of the LMM analysis appear in Table 4, including the β, SE, t, df, and p for VD and Pupil and for each of the 4 experiments (including the first Pilot experiment). Note the smaller *p*-values for the pupil measures compared to the VD, although all measures were highly significant.

**Table 4:** LMM statistical detail for Figure 4.

**Experiment**	β		**SE**		***t***		**df**		***p***	
	**VD**	**Pupil**	**VD**	**Pupil**	**VD**	**Pupil**	**VD**	**Pupil**	**VD**	**Pupil**
Pilot	0.025	−0.004	0.009	0.001	2.82	−5.77	478	1,948	0.005	8.9 × 10^−9^
Exp 1	0.033	−0.010	0.011	0.002	2.97	−4.88	978	877	0.003	1.2 × 10^−6^
Exp 2	0.035	−0.009	0.013	0.002	2.74	−4.02	387	376	0.006	7.0 × 10^−5^
Exp 3	0.049	−0.012	0.011	0.002	4.58	−6.11	502	473	5.8 × 10^−6^	2.1 × 10^−9^

### Exploratory pupillary measures of individual differences in VD

Both discomfort ratings and pupillary responses varied systematically with stimulus characteristics (Figure 2). To examine whether pupillary measures could capture individual differences in visual sensitivity, we conducted exploratory analyses in Experiment 1, which provided the largest sample size.

**Figure 5:**
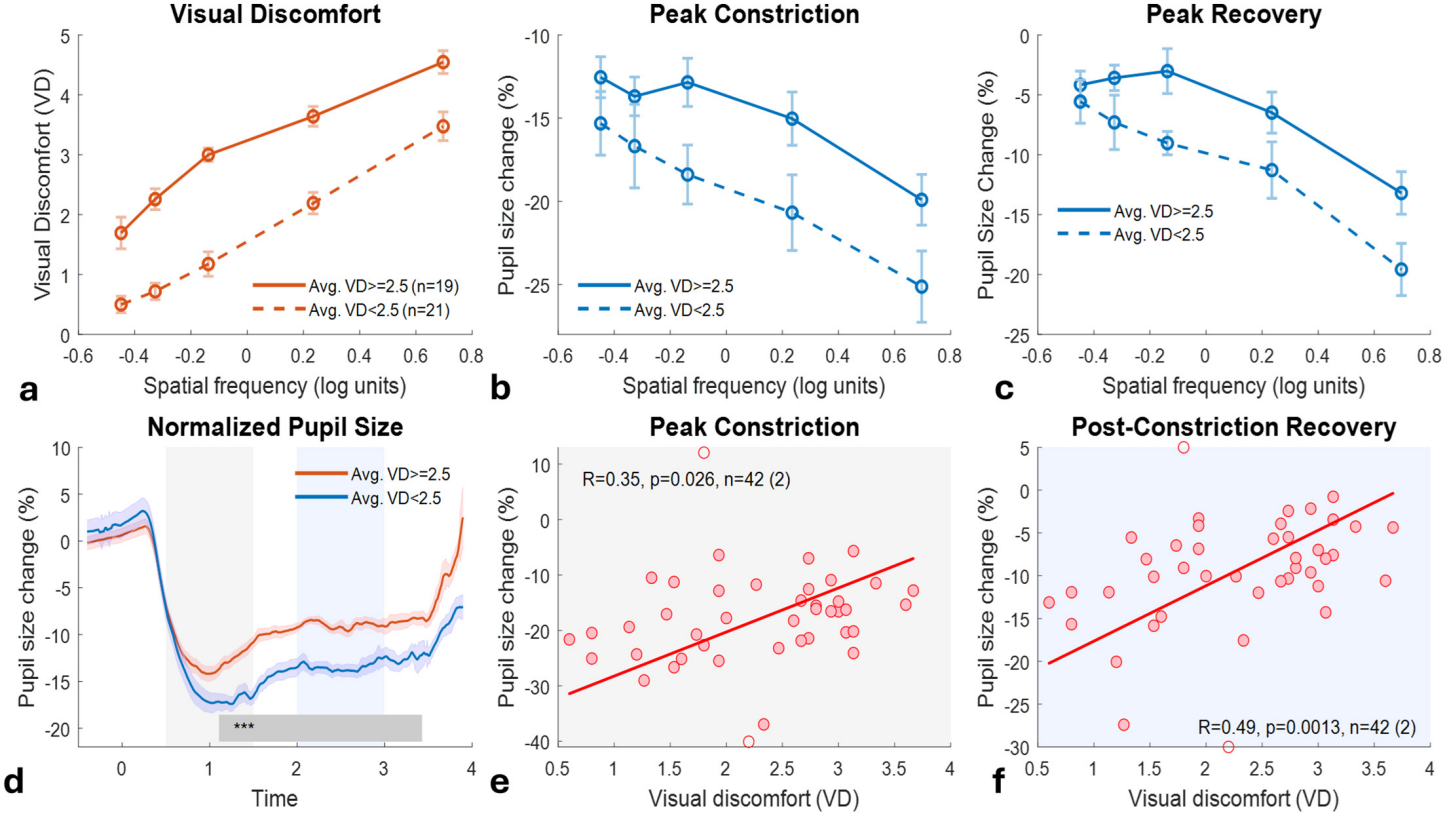
Pupillary responses and visual discomfort across spatial frequencies: group differences and individual correlations. (Top row) Group comparisons across five spatial frequencies [log10(freq) = −0.45 to 0.70, corresponding to 0.36–4.97 cycles per degree, cpd]. Participants were divided into high visual discomfort (Avg. VD ≥ 2.5, orange, *n* = 20) and low visual discomfort (Avg. VD < 2.5, blue, *n* = 22) groups based on average ratings. The number of participants per frequency bin varied slightly (18–20 per bin for both groups across measures) due to data quality filtering. **(a)** Visual discomfort ratings increase with spatial frequency in both groups, with the high-VD group consistently reporting greater discomfort. **(b)** Peak pupillary constriction shows frequency-dependent modulation, with stronger constriction (more negative values) at higher spatial frequencies in both groups. The high-VD group exhibits consistently weaker constriction responses across all frequencies. **(c)** Post-constriction pupillary recovery demonstrates frequency-dependent modulation, with weaker recovery (more negative values) at higher spatial frequencies. The high-VD group exhibits consistently stronger recovery (less negative values) across all frequencies. (Bottom row) Temporal dynamics and individual-level relationships. **(d)** Normalized pupillary response time courses (% change from pre-stimulus baseline). Gray shading (0.5–1.5 s) indicates the initial constriction phase; blue shading (2–3 s) indicates the recovery phase. Significant group differences emerge during the recovery period (1.1–3.4 s post-stimulus, *p* < 0.001, permutation test). **(e)** Individual-level correlation: higher average VD ratings are associated with weaker peak constriction (*R* = −0.35, *p* = 0.026, *n* = 42). **(f)** Individual-level correlation: higher average VD ratings are associated with stronger post-constriction recovery (*R* = 0.49, *p* = 0.0013, *n* = 42). Error bars represent standard error of the mean. All analyses restricted to viewing distance 75–85 cm. Statistical significance for time-series data assessed with permutation testing (1,000 iterations). Outlier removal for scatter plots performed at 1.33 SD.

A systematic scan of viewing distance ranges revealed that pupillary-discomfort correlations emerged only when participants maintained distances of 75–85 cm (*n* = 42 of 70). After outlier removal, we compared two groups: high-VD participants (average VD ≥ 2.5, *n* = 20) and low-VD participants (average VD < 2.5, *n* = 22). Both groups showed frequency-dependent patterns: VD ratings and peak constriction increased with spatial frequency (Figures 5a, b). However, the groups differed markedly in their response profiles. The high-VD group reported consistently greater discomfort across all frequencies (Figure 5a), yet exhibited weaker pupillary constriction (Figure 5b) and stronger post-constriction recovery (Figure 5c) compared to the low-VD group.

To quantify group differences statistically, we conducted a two-way ANOVA with Group (high-VD vs. low-VD) as a between-subjects factor and Spatial Frequency (5 levels, in log units) as a within-subjects factor. The results are shown in Table 5. For all dependent variables (VD, Figure 5a; Peak Constriction, Figure 5b; Peak Recovery, Figure 5c) there were significant effects of Group and log-Frequency, and a non-significant Group × log-Frequency interaction.

**Table 5:** Two-way ANOVA statistical detail for Figure 5.

**Dependent variable**	**Source of variation**	**df**	**F**	***p***	**η2**
VD (Figure 5a)	Group	1, 181	146.48	< 0.001^***^	0.447
	Spatial frequency	4, 181	79.67	< 0.001^***^	0.638
	Group × frequency	4, 181	1.26	0.287	0.027
Peak constriction (Figure 5b)	Group	1, 180	14.68	< 0.001^***^	0.075
	Spatial frequency	4, 180	6.83	< 0.001^***^	0.132
	Group × frequency	4, 180	0.31	0.874	0.007
Peak recovery (Figure 5c)	Group	1, 180	15.31	< 0.001^***^	0.078
	Spatial frequency	4, 180	13.98	< 0.001^***^	0.237
	Group × frequency	4, 180	0.62	0.649	0.014

Group refers to High vs. Low VD. ^***^Statistical significance.

Individual-level correlations confirmed this pattern: higher average discomfort was associated with weaker peak constriction (R = −0.35, *p* = 0.026; Figure 5e) and stronger recovery (R = 0.49, *p* = 0.0013; Figure 5f). Overall, in Experiment 1, which manipulated spatial frequency, higher visual discomfort was consistently linked to weaker pupillary constriction and stronger post-constriction recovery, both in group comparisons and in individual-level correlations.

## Discussion

### Systematic effects across experimental manipulations

Our findings demonstrate robust stimulus–response relationships across all experimental manipulations. Both visual discomfort (VD) ratings and pupillary constriction showed highly significant correlations with pattern characteristics (all *p* < 0.005) across experiments manipulating spatial frequency, central field size, and surround field size (Figure 2). The consistency of these relationships, despite variations in background colors and presentation durations, establishes spatial frequency and pattern area as fundamental drivers of both subjective and physiological responses to visual patterns.

### Dissociating luminance effects from spatial structure processing

A concern was raised that differences in stimulus luminance might account for the observed pupillary responses, making it difficult to confirm the relationship between visual discomfort and pupil size. Several aspects of our data directly address this issue and demonstrate that pupillary responses reflect spatial structure processing beyond simple luminance-driven reflexes.

First, across both the preliminary experiment and Experiment 1, pupillary responses exhibited patterns consistent with the Pupil Grating Response (PGR) (Barbur and Thomson, 1987; Sahraie et al., 2013), supporting recent findings Hu et al. (2019) that the responses reflected spatial-structure processing rather than luminance alone.

In the preliminary experiment, the pupil showed maximal dilation for the single black bar (lowest spatial frequency), reflecting the transition from white to reduced luminance. However, as spatial frequency increased, this dilation progressively diminished and reversed to net constriction at the highest frequency, despite similar mean luminance across conditions. In Experiment 1, where mean luminance was held approximately constant across all spatial frequencies, higher frequencies produced systematically stronger constriction, following the expected band-pass sensitivity to spatial frequency.

Second, experiments 2 and 3 provided a complementary luminance dissociation. In Experiment 2, increasing the patterned area reduced total screen luminance (from 274 to 137 cd/m^2^) yet produced stronger constriction, paralleling findings by Gao et al. (2020) showing that pupil constriction increases with field size even when overall luminance is held constant. In contrast, in Experiment 3, enlarging the central blank region increased luminance (from 137 to 214 cd/m^2^) yet produced weaker constriction and lower discomfort. The opposite relationship between luminance and pupillary response across these two experiments indicates that spatial configuration, not brightness, is the primary driver of the effects observed here.

This interpretation is further supported by evidence that pupillary responses are modulated by perceived brightness rather than physical luminance alone, as demonstrated in studies using brightness illusions and images of natural light sources (Laeng and Endestad, 2012). Together, these findings establish that pupillary constriction can increase with higher spatial frequency or larger pattern area, even when physical luminance remains constant or decreases.

Finally, the association between pattern viewing and pupillary constriction aligns with evidence that aversive visual stimuli recruit parasympathetic pathways (Ayzenberg et al., 2018). Interestingly, our observation that heightened sensitivity in the High-VD group was associated with weaker constriction resembles the recent findings of Qiu et al. (2025). They reported that while misophonic visual cues generally elicit constriction, this response was attenuated in the misophonic group compared to controls.

### Accumulation rather than adaptation

Repeated exposures produced progressive increases in discomfort ratings (Figure 4, left) and decreases in pre-stimulus pupil size (Figure 4, right). This pattern represents a departure from typical visual adaptation, where responses usually weaken with repetition. For example, contrast adaptation in the visual system shows weakening over time (Ohzawa et al., 1982), which serves as a classic demonstration of visual adaptation. In contrast, our findings indicate cumulative strain, similar to cumulative effects observed in pain research (Bar-Shalita and Cermak, 2015). Notably, our experiments did not include a separate familiarization session; instead, familiarization occurred during the very first trial, which likely explains why the cumulative effect became statistically significant only after excluding trial 1. In the central field size experiment, excluding near-floor conditions revealed a robust effect (p shifting from 0.55 to 0.0064). This suggests that cumulative discomfort arises only when stimuli exceed a threshold of aversiveness. These results also have implications for experimental design. Repeated stimulus presentations, often used to increase statistical power, may themselves alter the responses being measured, as subjective discomfort and pupil size both accumulate over time.

### Distinguishing cumulative visual stress from boredom and drowsiness

We consider the possibility that a progressive decrease in baseline pupil size reflects boredom or drowsiness rather than cumulative visual stress. Indeed, such states are well-documented causes of pupil constriction in repetitive psychophysical experiments (Unsworth and Robison, 2016). However, several aspects of our data suggest that these factors alone cannot fully account for the pattern of results. While boredom and drowsiness reduce pupil size, these states typically do not produce systematically increasing subjective discomfort ratings. Studies of boredom in repetitive tasks generally show decreased engagement, increased response variability, and reduced reporting effort (Pattyn et al., 2008), rather than the progressive, systematic increases in discomfort that we observed. We acknowledge that general arousal and fatigue are likely to contribute to pupil changes. However, the stimulus-specific effects and paradoxical individual differences (Figure 5) suggest that visual stress mechanisms play a central role. This interpretation aligns with established findings in sensory modulation research, where repeated exposure to aversive stimuli can produce cumulative sensitization rather than habituation (Bar-Shalita and Cermak, 2015).

### Individual differences and the sensitivity paradox

At the group level, stimuli that produced higher discomfort ratings consistently elicited stronger pupillary constriction (Figure 2). However, across individuals, a counterintuitive pattern emerged: those reporting greater overall discomfort showed weaker stimulus-evoked constriction and stronger late-phase redilation (Figure 5). As shown in Figures 5b–f, high-sensitivity participants maintained larger baseline pupil diameters and more pronounced recovery. This dissociation between subjective and physiological measures represents a paradox of sensitivity, suggesting that individuals who experience stronger discomfort may exhibit altered autonomic balance rather than simply amplified responses.

The most direct clinical parallel to this pattern is found in migraine photophobia, a form of light-induced visual discomfort. Cortez et al. (2017) investigated the pupillary light response (PLR), a reflex to a pure light stimulus, and found that higher interictal photophobia was associated with a mixed autonomic dysfunction, specifically involving impairments in both pupillary constriction (parasympathetic mechanism) and the late redilation phase (sympathetic mechanism).

Our findings extend this principle to pattern-induced discomfort (Pattern Glare), a more complex visual stress that involves both luminance and high-spatial-frequency contrast. The observed pattern of weaker constriction and enhanced redilation in our high-discomfort group strongly mirrors the autonomic dysregulation reported in migraine, suggesting that the physiological basis for subjective visual sensitivity, whether triggered by pure light or structured patterns, may involve a common pathway of impaired autonomic regulation under visual load. This parallel supports the potential of pupillometry to serve as an objective marker for general visual hypersensitivity.

## Limitations

Several limitations should be acknowledged. First, we assumed that participants with higher discomfort ratings represent individuals with greater visual sensitivity, but this was not validated with standardized clinical measures. Future studies should combine pupillometry with validated sensitivity questionnaires and include clinical populations. Second, accommodation may have influenced responses. Participants who sat very close to the screen tended to report stronger discomfort and greater pupillary constriction, raising the possibility that accommodative effort contributed to these effects. However, within the 75–85 cm range accommodation effects were minimized, and no consistent relationship was found between viewing distance, pupil responses, or discomfort. Haigh et al. (2013) reported that uncomfortable patterns did not modulate accommodation, and suggested that weaker accommodation may be a consequence of visual discomfort or a coping mechanism rather than its cause. Xu et al. (2015) showed that accommodative responses decrease as spatial frequency increases. Finally, we did not use a chinrest. This was intentional, to evaluate feasibility in populations such as children and to allow *post-hoc* filtering of viewing distances rather than enforcing rigid positioning. As a result, for the analysis of individual differences in visual discomfort, we excluded participants whose viewing distance frequently fell outside the reliable 75–85 cm range, reducing the effective sample size for the data in Experiment 1 from *n* = 70 to *n* = 42. Nevertheless, within this controlled range, our results were consistent with prior literature such as Cortez et al. (2017).

## Future research directions

Future research can extend the present findings in several directions. A natural next step is to examine how chromatic properties influence visual discomfort and pupillary responses when spatial frequency and field size are held constant. While the current study used achromatic black patterns on a constant brown or white background, previous work has shown that color strongly modulates pattern-induced discomfort even when spatial structure remains unchanged (Wilkins and Nimmo-Smith, 1987; Wilkins and Evans, 2010). Manipulating background chromaticity while controlling other parameters could enhance our ability to characterize individual visual sensitivity profiles, a direction we are currently pursuing. Clinically, future work should test whether the pupillary and discomfort patterns observed here generalize to populations with known visual hypersensitivities, such as migraine, post-concussion, or photosensitivity syndromes. Our finding that individuals reporting greater discomfort exhibited weaker pupillary constriction parallels results in migraine (Cortez et al., 2017), suggesting that atypical pupillary modulation could serve as a noninvasive biomarker of visual hypersensitivity. Finally, these lines of work may ultimately support the development of objective screening tools and practical assessments for individuals with heightened visual sensitivity.

## Conclusions

This study systematically investigated the effects of square-wave gratings varying in spatial frequency or area on visual discomfort and pupillary dynamics, examining both stimulus-specific responses and cumulative trends across repeated trials. Three primary findings emerged. First, visual discomfort ratings and pupillary constriction increased systematically with higher spatial frequencies and larger pattern areas. Importantly, this relationship held even when luminance was controlled or inversely manipulated, indicating that pupillary responses and subjective ratings were associated with the spatial structure rather than luminance alone. Second, repeated exposures produced progressive increases in discomfort ratings and decreases in baseline pupil size. While non-visual factors such as drowsiness or fatigue may contribute to pupil reduction over time, the concurrent increase in subjective discomfort suggests accumulation rather than simple adaptation. This implies that while repetition is standardly used to enhance statistical reliability, in this context it may alter the measured state, requiring assessment protocols to account for these cumulative effects. Finally, individual differences revealed a paradoxical pattern: participants reporting the greatest overall discomfort exhibited weaker stimulus-evoked constriction and stronger post-stimulus recovery. This dissociation resembles autonomic patterns observed in migraine photophobia and suggests that visual hypersensitivity involves altered autonomic control rather than simple sensory amplification. From a clinical perspective, these findings support the potential of pupillometry as an objective, non-invasive complement to current subjective tools for visual sensitivity, particularly for non-verbal populations.

## Data Availability

The raw data supporting the conclusions of this article will be made available by the authors, without undue reservation.
